# Genetic polymorphisms of human cytochrome P450 CYP1A1 in an Egyptian population and tobacco-induced lung cancer

**DOI:** 10.1186/s41021-016-0066-4

**Published:** 2017-01-07

**Authors:** Nada Ezzeldin, Dalia El-Lebedy, Amira Darwish, Ahmed El-Bastawisy, Mirhane Hassan, Shereen Abd El-Aziz, Mohamed Abdel-Hamid, Amal Saad-Hussein

**Affiliations:** 1 Chest Diseases, National Research Center, Cairo, Egypt; 2 Department of Clinical and Chemical Pathology, National Research Center, Cairo, Egypt; 3 Medical Oncology, National Cancer Institute, Cairo University, Cairo, Egypt; 4 Medical Molecular Genetics, National Research Center, Cairo, Egypt; 5 Department of Environmental Health and Preventive Medicine, National Research Center, Cairo, Egypt

**Keywords:** Lung cancer, *CYP1A1*, Polymorphisms, Tobacco smoking

## Abstract

**Background:**

Cytochrome P450 CYP1A1 helps detoxify the potential carcinogens in tobacco smoke, it was reported that polymorphisms in the coding gene result in variation in the expression and activity levels which alter metabolism and clearance of carcinogens and therefore modify cancer risk. In this work, we aimed to identify *CYP1A1* gene polymorphisms associated with lung cancer in Egyptian population and to examine the interaction effect with Tobacco smoking in modulating disease risk.

**Methods:**

A case–control study was conducted on 150 unrelated lung cancer patients and 150 unrelated control subjects. Genomic DNA was extracted and sequencing analysis of *CYP1A1* gene was performed on ABI PRISM 3100 genetic analyzer.

**Results:**

Three variants in *CYP1A1* gene were identified in heterozygous forms in lung cancer patients *I462V, T461N* and *I286T*. A combined variant *T461N/ I462V* associated with lung cancer and those who carried this variant were 2-times more likely to develop lung cancer (OR = 2.03, 95% CI = 1.81-2.29, *P* = 0.04), specially the non-small cell type (NSCLC) (OR = 2.20, 95% CI = 1.93–2.50, *P* = 0.02). Wild type was more frequent among smoker controls (83.3%) compared to smoker lung cancer patients (54.8%), *P* = 0.03. Association studies to examine the interaction effect of identified variants with Tobacco smoking in modulating disease risk showed no significant associations. Identified polymorphisms showed no significant implication on the stage or the prognosis of the disease.

**Conclusion:**

Our findings support that *CYP1A1* polymorphisms play a role in the pathogenesis of lung cancer. In Egyptian population, *CYP1A1 I462V, T461N and I286T* variants were identified among lung cancer patients and combined *T461N/ I462V* was a risk variant for NSCLC in non smokers.

## Background

Lung cancer progression is characterized by cumulative alterations in key molecules involved in the cell cycle, signaling and angiogenesis pathways. Most lung cancer patients demonstrate chromosomal abnormalities at the site of tumor suppressor genes or have mutations in known oncogenes [1].

In the laboratory, chemicals in tobacco reduce the capacity to repair DNA damage in cells from lung cancer patients than cells from normal individuals [2]. As many smokers do not develop lung cancer, it is likely that inherited factors influence the effects of tobacco. There is a considerable interest in variants of genes that help detoxify the carcinogens in tobacco smoke, such as members of the cytochrome P450 (*CYP*), glutathione S-transferase (*GST*) and N-acetyltransferase (*NAT)* gene families [3].

Scientists have identified 57 human *CYP* genes and 33 pseudo genes divided into 18 families and 42 subfamilies. Now *CYP1A1, CYP1A2, CYP1B1, CYP2A6, CYP2C9, CYP2C19, CYP2D6, CYP2E1, CYP3A4, CYP3A5* and other gene polymorphisms have been confirmed and specific metabolic enzyme phenotypes differ with regions and races [4]. *CYP* gene changes can cause increased activity of the enzyme, decreased activity or even inactivity. In addition, mutations at the substrate recognition sites may lead to changes in enzyme specificity [5].

CYP1A1 plays a major role as a carcinogen activating enzyme within the CYP system. Unlike most CYP enzymes, CYP1A1 expression is mainly found in extra hepatic tissues, including the lung, where it metabolizes and is markedly induced by polycyclic aromatic hydrocarbons (PAHs) [6]. Elevated CYP1A1 inducibility is associated with pulmonary PAH-related DNA adduction [7] and high lung cancer risk [8]. Both CYP1A1 expression and the formation of these PAH-DNA adducts in human lung tissue are highly variable [9–11], possibly due to differing exposure to environmental factors and to genetic polymorphisms affecting the *CYP1A1* gene locus [12].

The first variant allele identified was CYP1A1*2A (MspI or m1 polymorphism) and is found in 5% of Caucasians [13]. CYP1A1*2C (Ile462Val or m2 polymorphism) is rare in Caucasians and is usually detected with CYP1A1*2A [14]. The combination of both variants is referred to as CYP1A1*2B. CYP1A1*3, consisting of a T3205C base change (m3), seems to show enhanced enzyme activity, although it is extremely uncommon in Caucasians [13]. Finally, CYP1A1*4, a Thr461Asn (m4) amino acid change, detected in Caucasians with a frequency of roughly 3%, has also been related to greater enzyme catalytic efficiency [15]. These *CYP1A1* polymorphisms have been extensively studied with regard to risk of lung cancer. However, whereas some studies report increased risk in the presence of some of the mutations [16, 17], there are many other contradictory results due to ethnic differences [18, 19].

The aim of this work is to identify CYP1A1 gene polymorphisms associated with lung cancer in Egyptian population and to examine the interaction effect with Tobacco smoking in modulating disease risk.

## Methods

### Subjects

This work was collaboration between National Cancer Institute (NCI) and National Research Center (NRC), Cairo, Egypt. A case–control study was conducted on 150 unrelated adult patients with primary lung cancer and 150 unrelated controls. Patients were presented to NCI from different governorates of Egypt; Cairo, Giza, Qalyubia, Sharqia, Monufia, Kafr El-Sheikh, Minya, Faiyum, Asyut, Sohag and Qena. All subjects included in the study were interviewed to fill a medical questionnaire with special consideration to the lifetime history of tobacco use, residence, occupational history and family history of cancer. Thorough clinical examination and chest radiography were applied. Blood sample was obtained from each subject for sequencing analysis of the human CYP1A1 gene. Sample for histopathology examination of cancer was obtained from each patient either by open biopsy or via bronchoscopy. The exclusion criteria included previous history of cancer, metastasized cancer from other organs, patients with pulmonary fibrosis, acute interstitial pneumonia and previous radiotherapy or chemotherapy or receiving any anti-cancer treatment before enrollment in the study. The study was approved by the ethics committee of the National Research Center. All subjects were aware by the nature of the study and gave a written informed consent.

## Methodology

### DNA extraction

Genomic DNA was extracted from blood samples obtained from 150 controls and 150 lung cancer patients using QIAamp DNA extraction kit (Qiagen Hilden, Germany, Cat no. 51304) according to the manufacturer's instructions.

### Sequence analysis of the CYP1A1 gene

**Fig. 1:**
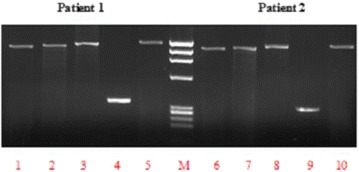
2% agarose gel illustrating the amplification of CYP1A1 gene in two patients. Lanes 1 and 6: amplification of exon 1 (1153-bp). Lanes 2 and 7: amplification of exon 2 (1180-bp). Lane 3 and 8: amplification of exons 3–6 (1233-bp). Lanes 4 and 9: amplification of exon 7a (349-bp). Lanes 5 and 10: amplification of exon 7b (1330-bp). M: Size marker (PhiX174 DNA/HaeIII digest)

The sequence of the human *CYP1A1* gene described in the GenBank (accession number X02612) was used as a reference. The primers used for the amplification and the direct sequencing of all seven exons and exon-intron junctions of the gene are shown in Table 1. PCR for each fragment of *CYP1A1* gene was conducted in a 25 μl reaction mixture containing 100 ng of genomic DNA, 20 pmol of each primer and 12.5 μl of AmpliTaq Gold 360 Master Mix (Applied BioSystems, Foster City, CA, USA). PCR cycling conditions consisted of initial denaturation at 94 °C for 5 min, followed by 30 cycles of denaturation at 94 °C for 30s, annealing at 60 °C for 30s and extension at 72 °C for 30s, followed by a final extension of 5 min at 72 °C. PCR products were checked first on 2% agarose gel for successful amplification (Fig. 1) and was further purified using PureLink Quick PCR Purification Kit (Invitrogen, Germany). The purified PCR products were directly sequenced in both directions using the Big Dye Termination kit (Applied Biosystems, Foster City, CA, USA) and sequences were determined using ABI PRISM 3100 genetic analyzer (Applied Biosystems).

**Table 1:** Primers used for amplification and direct sequencing analysis of the human CYP1A1 gene

Primers	Primer sequence (5′–3′ orientation)	Location	Amplified exon
1A1 ex1-S	CCGAGTCCTGGTAGGCTGTA	5′-fanking	Exon 1
1A1 ex1-A	CCTGCAGTTGGCAATCTGTC	intron 1	
1A1 ex2-S	CCCACAGTGGTAGTTCAACA	intron 1	Exon 2
1A1 ex2-A	CCCTGCCAAGGAAGAAGACT	intron 2	
1A1 ex3-S	AGAGCCTTGCAGAGGCAGAG	intron 2	Exons 3–6
1A1 ex6-A	GGCAATGGTCTCACCGATAC	exon 6	
1A1 I462V-S	GCTGCTTGCCTGTCCTCTAT	intron 6	Exon 7a
1A1 I462V-A	AGGCATGCTTCATGGTTAGC	exon 7	
1A1 ex7-S	AGCTATGGGTCAACCCATCT	exon 7	Exon 7b
1A1 ex7-A	TCTTCTTCCTCCCTACAGTA	intron 7

### Statistical analysis

Data were analyzed using SPSS version 18.0 (Chicago, IL, USA). Data were expressed as number and percentage of total for categorical variables. Chi-square test (χ^2^) was used to compare the distribution of CYP1A1 genotypes between groups. Likelihood ratio was used when the expected count was less than 5 in more than 20% of the cells. The associations between genotype and risk of lung cancer were estimated by odds ratio (OR) and 95% confidence interval (95% CI) using logistic regression models. The ORs were adjusted for age, smoking status, and pack-years. *P*-value <0.05 was considered significant.

## Results

The study included 300 subjects; 150 unrelated lung cancer patients and 150 unrelated control subjects. A statistical significant age difference was found between controls (mean 43.3 ± 11.1 years) and patients (mean 56.7 ± 9.79 years) (*p* < 0.001), denoting that older age is associated with higher risk of lung cancer. There was a statistical significant difference in gender between the two groups with 51.3% females and 48.7% males in control group vs. 24% females and 76% males in patients group (X^2^ = 25.82, *P* < 0.0001), with 3.5-times increased risk to develop lung cancer in males than in females (OR = 3.49, CI: 2.13–5.69).

Mean pack-years was significantly higher in patients (21.4 ± 2.25) than in controls (4.0 ± 0.77). Smoking habit was significantly higher among patients (X^2^ = 56.88, *P* < 0.0001), with an odd ration of 6.69 (CI: 3.99–11.20) denoting a higher risk to develop lung cancer in smokers by 6.7 times the non-smokers.

**Fig. 2:**
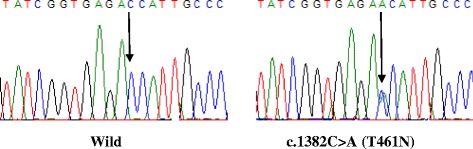
Portion of the sequencing phoregram of exon7 of CYP1A1 gene showing the c.1382C > A (T461N) polymorphism. The *arrow* indicates the site of the variant nucleotide position

**Fig. 3:**
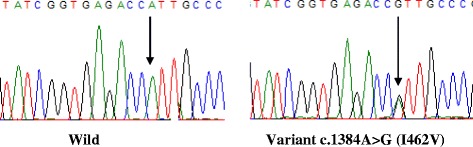
Portion of the sequencing phoregrams of exon 7 of CYP1A1 gene showing the c.1384A > G (I462V) polymorphism. The *arrow* indicates the site of the variant nucleotide position

**Fig. 4:**
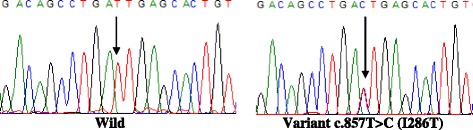
Portion of the sequencing phoregram of exons 3–6 of CYP1A1 gene showing c.857 T > C (I286T) polymorphism found in only one subject. The *arrow* indicates the site of the variant nucleotide position

Our sequencing analysis of *CYP1A1* gene identified 2 variants in exon7 in control subjects; CYP1A1*4 1382C > A (T461N) (*n* = 33) (Fig. 2) and CYP1A1*2C 1384A > G (I462V) (*n* = 19) (Fig. 3). In lung cancer patients, 3 variants were identified, CYP1A1*2C (*n* = 17), CYP1A1*4 (*n* = 46) and combined CYP1A1*2C/ CYP1A1*4 (*n* = 4). The very low frequent variant in the database CYP1A1 857 T > C (I286T), which was found only 2 times in thousands of human genomes, has been detected in exons 3–6 of the *CYP1A1* gene in a non-smoker adenocarcinoma case (Fig. 4). All identified variants were detected in the heterozygous form.

There was no significant difference between controls and lung cancer patients regarding the frequency of identified *CYP1A1* variants, except for the combined variants CYP1A1*2C/ CYP1A1*4 which associated with 2-times higher risk of lung cancer (OR = 2.03, 95% C.I:1.81–2.29, *P* = 0.04), specially the non-small cell type (NSCLC) (OR = 2.20, 95% C.I: 1.93–2.50, *P* value = 0.02) (Table 2) with no significant difference between the pathological subtypes of NSCLC (*P* > 0.05).

**Table 2:** Frequency of CYP1A1 variants among lung cancer patients and control

CYP1A1	Control^a^	Cases^a^	OR (95% CI)	NSCLC^a^	OR (95% CI)	SCLC^a^	OR (95% CI)
Variant	*n*=150	*n*=149^b^	*P*- value	*n*=129	*P* value	*n*=20	*P*- value
CYP1A1*2C	19(12.7)	17(11.4)	1.126(0.56–2.26) 0.7	13(10.1)	0.77(0.37–1.63) 0.4	4(20)	1.72(0.52–5.70) 0.3
CYP1A1*4	33(22)	46(30.9)	0.63(0.38–1.06) 0.08	41(31.8)	1.65(0.97–2.82) 0.06	5(25)	1.18 (0.40–3.49) 0.7
CYP1A1*2C/*4	0(0)	4(2.7)	2.03(1.81–2.29) 0.04^c^	4(3.1)	2.20(1.93–2.50) 0.02^c^	0(0)	–
CYP1A1*1 (wild type)	98(65.3)	82(55)	0.65(0.41–1.04) 0.07	71(55)	0.11(−0.01–0.23) 0.07	11(55)	1.54(0.60, 3.96) 0.3

*SCLC* small cell lung cancer, *NSCLC* non-small cell lung cancer

^a^Data presented as N (%)

^b^The case carrying rare CYP1A1 857 T > C (I286T) variant was excluded from the statistical analysis

^c^significant *p*

There was statistical significant difference in the distribution of *CYP1A1* variants in relation to smoking habit among lung cancer patients and control subjects. The wild type gene was the most frequent among smoker controls compared to non-smoker controls and lung cancer patients (*P* = 0.03) (Table 3). Association studies of *CYP1A1* polymorphisms and smoking with susceptibility to lung cancer showed no significant association with disease risk (Table 4). No significant association of *CYP1A1* polymorphisms and pack year with susceptibility to lung cancer (Table 5). Identified polymorphisms showed no significant implication on the stage or the prognosis of the disease (Tables 6 and 7).

**Table 3:** Frequency of CYP1A1 variants in relation to smoking habit in patients and control

CYP1A1 Variant		Control		Patients		Chi-square	*P*-value
		Non-smoker	Smoker	Non-smoker	Smoker		
		*N* = 120	*N* = 30	*N* = 56	*N* = 93		
CYP1A1*2C	N (%)	18 (15)	1 (3.3)	7 (12.5)	10 (10.8)	18.9	0.03^a^
CYP1A1*4	N (%)	29(24.2)	4 (13.3)	15 (26.8)	31 (33.3)		
CYP1A1*2C/*4	N (%)	0 (0)	0 (0)	3 (5.4)	1 (1.1)		
CYP1A1*1 (wild type)	N (%)	73 (60.8)	25 (83.3)^a^	31 (55.4)	51 (54.8)		

^a^Significant *P*

**Table 4:** Association of CYP1A1 polymorphisms and smoking with lung cancer risk

CYP1A1 Variant	Non-smokers	Smokers	OR	95% CI	*P*-value
	*N* = 56	*N* = 93			
	N (%)	N (%)			
CYP1A1*2C	7 (12.5)	10 (10.8)	1.19	(0.42–3.32)	0.75
CYP1A1*4	15 (26.8)	31 (33.3)	0.73	(0.35–1.52)	0.20
CYP1A1*2C/*4	3 (5.4)	1 (1.1)	5.21	(0.53–51.3)	0.12
CYP1A1*1 (wild type)	31 (55.4)	51 (54.8)	0.98	(0.50–1.91)	0.95

**Table 5:** Distribution of CYP1A1 polymorphisms in heavy (pack year ≥ 20) vs. light (pack year < 20) smoking in lung cancer patients and control

CYP1A1 variant		Smoking heaviness				Likelihood ratio	
		Control		patients			
		Light	heavy	Light	heavy	ratio	*P*-value
		*N* = 16	*N* = 14	*N* = 37	*N* = 56		
CYP1A1*2C	N (%)	0 (0)	1 (7)	1 (2.7)	9 (16.1)	15.86	0.07
CYP1A1*4	N (%)	1 (6.3)	3 (21.4)	12 (32.4)	19 (33.9)		
CYP1A1*2C/*4	N (%)	0 (0)	0 (0)	0 (0)	1 (1.8)		
wild type	N (%)	15 (93.8)	10 (71.4)	24 (64.9)	27 (48.2)

**Table 6:** Effect of CYP1A1 polymorphisms on lung cancer staging

CYP1A1 gene variant			Stage			Likelihood	*P*-value
			II	III	IV		
			*N* = 25	*N* = 44	*N* = 80		
	CYP1A1*2C	N (%)	19 (4)	5 (11)	11(14)	10.22	0.333
	CYP1A1*4	N (%)	9 (36)	15 (34)	22 (28)		
	CYP1A1*2C/*4	N (%)	1 (4)	0 (0)	3 (4)		
	CYP1A1*1 (wild type)	N (%)	14 (56)	24 (55)	44 (55)

**Table 7:** Effects of CYP1A1 polymorphisms on disease prognosis of available follow up cases

CYP1A1 variant	Response (n = 22)						Chi	*P*
	Complete response	Partial response	Progression	Refractory disease	Regression	Stable disease		
	N (%)	N (%)	N (%)	N (%)	N (%)	N (%)		
CYP1A1*2C	0 (0)	0 (0)	0 (0)	0 (0)	0 (0)	0 (0)	–	–
CYP1A1*4	0 (0)	0 (0)	2 (22.2)	0 (0)	1 (25.0)	1 (25.0)	6.8	0.33
CYP1A1*2C/*4	0 (0)	0 (0)	1 (11.1)	0 (0)	0 (0)	0 (0)	3.08	0.79
wild type	2 (100)	2 (100)	6 (66.7)	1 9(100)	3 (75.0)	3 (75.0)	12.2	0.052

## Discussion

*CYP1A1* is a polymorphic gene located at 15q24.1 with 7 exons and 6 introns. In addition to the wild-type (CYP1A1*1), 10 variant alleles have been identified [7]. Variants CYP1A1*2A, CYP1A1*2C, CYP1A1*3 and CYP1A1*4 with trivial names *m1, m2, m3 and m4*; respectively were the most commonly studied for cancer link [20, 21].

About 90% of lung cancer is strongly associated with Tobacco smoking [22]. Our results showed an estimated risk for lung cancer 6.7 times greater in Egyptian smokers than in non-smokers compared to 2.61 times in a previous study [23] and an increased risk to develop lung cancer in males by 3.5-times than in females, which might be attributed to the higher incidence of smoking habit among males.

In our patients, three polymorphisms in *CYP1A1* gene were identified; CYP1A1*2C (I462V) was detected in 12.7% of control subjects and 11.4% of patients, and CYP1A1*4 (T461N) identified in 22% of controls and 30.9% of patients. In one non-smoker adenocarcinoma patient, the rare *CYP1A1* 857 T > C (I286T) was identified. Ethnic difference in the distribution of CYP1A1*2C variant has been demonstrated in lung cancer patients, while few reports are available for CYP1A1*4. Frequencies of CYP1A1*2C ranged from 2.2% to 8.9% in Caucasians and was about 19.8% in Japanese, while CYP1A1*4 allele was found in 2% to 5.7% of a Caucasian population [24–27]. Asian reports rarely observed presence of CYP1A1*4 variant [21, 28, 29], meanwhile, it was more common among whites [30] and it was reported, by itself, as a lung cancer risk factor in Caucasians [12].

CYP1A1*2C and *4 variants have generally been associated with moderate to high risk of lung cancer [21]. These variants are rare in Caucasians and African-Americans. While studies of African-Americans have reported predominantly negative findings [31, 32], studies of Caucasians have been mixed [33, 34]. Although I462V polymorphism is relatively frequent in Asian populations (18% to 25%), the Val allele is rare in Caucasian control populations, occurring in 7% to 13% of people [35–37] which agreed with obtained results in our control population (12.7%).

In the studied Egyptian population, combined variant T461N/ I462V (m2/m4) was found in 2.7% of lung cancer patients associated with 2-times higher risk to develop lung cancer compared to either control or individual m2 or m4 variant carriers.

In an Australian study to identify lung cancer-risk modifying *CYP1A1* haplotypes, *2A and *2C variants were significantly over-represented in NSCLC cases compared to controls, whereas *4 variant was under-represented. *CYP1A1* haplotypes (in allele order CYP1A1*4, *2C, *2A); CGC and CG associated with increased risk of lung cancer confirming CYP1A1 polymorphisms as minor risk factor for NSCLC. It was reported that CYP1A1*2C increase the overall risk of NSCLC with an odd ratio of 2.88 (95% CI = 1.70–5.00, *p* < 0.001) [38], in accordance with our results in which CYP1A1*4 and *2C associated with increased risk of NSCLC by 2 times. On the other hand, San Jose et al. reported that I462V and T461V increase the risk to lung cancer in Spanish population, especially to SCLC [12]. Hung et al. found that I462V associated with higher risk for lung cancer, especially for lung adenocarcinoma [39]. Song et al. noted significantly higher risk of lung cancer for I462V variant allele, even in the heterozygous form. However, this elevated risk was restricted to squamous cell carcinoma only, not for adenocarcinoma or other histological types of lung cancer [21]. In our study, no significant difference of lung cancer risk for variant alleles between different pathological subtypes of NSCLC. We could not demonstrate a role of CYP1A1on the stage or the prognosis of the disease, in agreement with a previous study in Taiwan [40].

Controversial results were obtained from previous studies on interaction of smoking and *CYP1A1* variants. In the present study CYP1A1*1, wild type, was statistically more frequent among smoker controls. There was no association demonstrated between the heterozygous alleles of *CYP1A1* variants, smoking or smoking heaviness in prediction of lung cancer, 75% (3 of 4) of *CYP1A1* combined variant (T461N/ I462V) carriers were non-smokers.

Smoking was identified as a predominant risk factor and CYP1A1*2A polymorphism significantly associated with increased lung cancer risk (OR = 1.69; 95% CI = 1.11–2.59, *p* = 0.01), whereas CYP1A1*2A and *2C and Ile105Val imparted increased risk in non-smokers only [41]. Though, Wenzlaff et al. found no significant association with any of *CYP1A1* variants in never smokers [42], a recent pooled analysis by Hung et al. reported more than 2-fold increase in lung cancer susceptibility for both CYP1A1*2B and *2C variants among non-smoker Caucasians [39]. According to Song et al., non-smokers with CYP1A1*2A variant had elevated risk than those homozygous for wild type alleles [21]. Previous study in life-time non-smoking Chinese women reported an elevated risk of lung cancer for both CYP1A1*2B and *2C homozygous genotypes, furthermore, lung cancer risk associated with both polymorphisms was higher in women with lower environmental tobacco smoke exposure [43]. I462V polymorphism is not related to lung cancer overall, but it might play a role at lower levels of Tobacco smoking among subjects with impaired carcinogen detoxification [44].

## Conclusion

Beside the wild type CYP1A1*1, three variants of CYP1A1 gene were identified in Egyptian population; CYP1A1*2C, CYP1A1*4 and the rare *CYP1A1 c.857 T > C* (*I286T*). Combined variant CYP1A1*2C/ CYP1A1*4 associated with higher risk of lung cancer specially NSCLC among non-smokers. Identification of these variants may help in risk assessment, early detection and improvement of current treatment options for lung cancer patients. Further studies to clarify the role of these variants in the pathogenesis of the disease are needed.

## Abbreviations

Asn: Asparagines
CYP: Cytochrome P450
GST: Glutathione S-transferase
Ile: Isoleucine
NAT: N-acetyltransferase
NSCLC: Non-small cell lung cancer
PAH: Polycyclic aromatic hydrocarbons
SCLC: Small cell lung cancer
Thr: Threonine
Val: Valine
